# Composition and diversity of soil bacterial communities under identical vegetation along an elevational gradient in Changbai Mountains, China

**DOI:** 10.3389/fmicb.2022.1065412

**Published:** 2022-12-01

**Authors:** Mengsha Li, Guanhua Dai, Liqiang Mu

**Affiliations:** ^1^School of Forestry, Northeast Forestry University, Harbin, China; ^2^Institute of Nature and Ecology, Heilongjiang Academy of Sciences, Harbin, China; ^3^Research Station of Changbai Mountain Forest Ecosystems, Chinese Academy of Sciences, Erdaobaihe, China

**Keywords:** soil nutrients, bacterial diversity, temperate mountain forest, diversity index, soil bacteria

## Abstract

Soil bacteria play important roles in biogeochemical cycling and biodiversity in mountain ecosystems. Past studies have investigated the bacterial community composition and diversity in elevation gradations covered by different vegetation types, but for a better assessment of elevation effects, here we studied bacterial communities in soil under identical vegetation cover. High-throughput amplicon sequencing of the V3-V4 region of bacterial 16S rDNA was used to investigate the diversity and composition bacterial communities in soil from 700 to 1,000 m above sea level collected on the north slope of Changbai Mountains, Northeast China. Obviously differences (*p* < 0.05) in soil physicochemical parameters (i.e., total nitrogen, nitrate and ammonium nitrogen, soil moisture content, available potassium, microbial biomass carbon and nitrogen) were observed at different elevations. Soil bacterial abundance indices (Richness, Chao1, ACE) differed significantly along the elevation gradient, whereas the Shannon index remained unchanged. Principal Coordinates Analysis indicated separated soil bacterial communities of the different elevations. The dominant phyla in all soil samples were Proteobacteria, Acidobacteria, Actinobacteria, Verrucomicrobia, and Bacteroidetes, which in combination reached 80%–85%. Soil pH to some extend related to soil bacterial community along altitude gradations. The relative abundance of a multiple phyla was negatively affected by the soil nutrients, such as ammonium and nitrate nitrogen, available potassium, soil moisture content, available phosphorus, microbial biomass nitrogen and soil organic C. The strongest effects were seen for Proteobacteria. The pH either positively or negatively correlated with specific genera. The soil bacterial function differed significantly among four elevations. The chemoheterotrophy, aerobic chemoheterotrophy and nitrification were the most dominant functions of soil bacteria among four elevations. Overall, the changes in soil physicochemical properties with elevation are important in shaping the bacterial diversity, composition and function in soil with the same above-ground vegetation of Changbai Mountains.

## Introduction

Soil bacteria are an important component of the soil microbiota and play crucial roles in regulating ecological functions such as litter decomposition, biogeochemical cycling, and maintaining biodiversity (Frey et al., 2016). Since bacteria are sensitive to environmental changes, they can be considered as important indicators for assessing soil nutrient cycling and maintaining ecosystem balance (Shen et al., 2013; Deng et al., 2019). Elevation gradients provide geographic variation that can coincide with changes in environmental factors such as temperature, precipitation, vegetation type, and soil properties (Ren et al., 2021). These represent a natural experimental platform for assessing how soil bacterial communities respond to environmental change (Körner, 2007). There remains controversy regarding the patterns of changes in soil bacterial diversity and community structure along an altitudinal gradient, whose plot can produce declining (Nottingham et al., 2018), depressed (Shen et al., 2020), humped (Ren et al., 2018) or stepped curves of soil bacterial diversity along the altitudinal gradient. Soil bacterial community composition can differ significantly at different altitudes, but opposing results have been reported (Singh et al., 2014; Li et al., 2018). The functions of soil bacterial ecosystems are closely related to the structural characteristics of their communities (Bardgett and Van Der Putten, 2014). Shen et al. (2016) found that the abundance of soil bacterial functional genes in samples taken from the Changbai Mountains, China, tended to increase with elevation. An et al. (2021) also showed that the metabolic pathway genes of soil bacteria in the Sedi La Mountains, China, significantly changed along the altitude gradient and identified a strong correlation between the structure and function of the bacterial community. In general, the activity and structure of soil microbial communities and their diversity are influenced by a combination of biotic and abiotic factors that include plant species and the soil environment (Weng et al., 2021; Sui et al., 2022). At present, most available studies have focused on elevational gradients where there are large differences between vegetation and local ecosystem, which hampers interpretation of the results. The distribution patterns of bacterial community characteristics (diversity, structure and function) within ecosystems at different elevations but with the same vegetation type remain to be explored. Furthermore, there is uncertainty about how the structure and function of soil bacterial communities are related to each other.

Bacterial community composition significantly corresponds to vegetation and habitat selection (Chang et al., 2020), and therefore the composition and diversity of bacterial communities are strongly influenced by the composition of above-ground vegetation and soil environmental conditions (Sui et al., 2021). Previous studies have shown that changes in vegetation type and plant biomass on elevational gradients impact bacterial community diversity and structure by altering soil nutrient inputs (Xu et al., 2014). The vegetation type along an elevation can also affect the local input of organic matter into the soil and this potentially alters the soluble organic carbon content of soils, which in turn has a significant impact on the structure and function of soil bacterial communities (Shen et al., 2016). However, Carney and Matson (2006) showed that plant diversity only had a weak effect on soil microbial biomass. Consequently, the complexity of above-ground ecosystem species makes the interpretation of below-ground ecosystems more challenging (Bardgett et al., 1998). It can be expected that changes in vegetation type result in a more complex response of soil bacterial community characteristics to elevation.

The Changbai Mountains in the northeast of China represent an area with rich and well-preserved subtropical biodiversity gene pools at the same latitude. The area is sensitive to climate changes and is a current hotspot for research (Ping et al., 2017). Several studies have been conducted on the effects of soil microbial diversity and community composition at different altitude gradients in Changbai Mountains. For example, Han et al. (2018) compared the soil of broadleaf Korean pine forests at different altitudes and found that the composition and diversity of the bacterial communities were mutually influenced by soil physicochemical properties and the composition of above-ground vegetation. Zhang et al. (2022) reported that soil microbial community structure and enzyme activity in the vertical zone of the Changbai Mountains were closely related to vegetation community composition, soil environmental factors, and hydrothermal conditions. Yang et al. (2017) showed that different altitudinal gradients in Changbai Mountains affected above-ground vegetation composition, leading to changes in soil fungal communities and diversity through plant litter. Clearly, current research on soil microorganisms at different altitude gradients in the Changbai Mountains has been influenced by the composition of the above-ground vegetation, and the effect of above-ground vegetation composition on soil microorganisms could not be eliminated. The effect of different elevation gradients on soil microorganisms under the same conditions of above-ground vegetation remains to be determined.

The deciduous tree *Tilia amurensis* Rupr. is dominant at a wide altitude distribution on the northern slopes of Changbai Mountains (Kang et al., 2021). It is an important species in the Korean pine forests and plays important roles in biogeochemical cycles, biodiversity, and other ecological functions and services of Korean pine forest ecosystem. The main distribution elevations are from 700 to 1,000 m in Changbai Mountain. Therefore, this supply an ideal field platform for us to investigate the distribution regulation of soil microbial diversity, composition and structure along with elevation gradations. So, this study investigated soil sampled between 700 m and 1,000 m altitude from the northern slopes of Changbai Mountains where the vegetation composition was identical (i.e., *T. amurensis*). The questions addressed were: (1) How do the soil bacterial community characteristics (diversity, structure and function) change along an altitudinal gradient under the same vegetation? (2) What are the driving factors for the elevational distribution patterns of soil bacterial community characteristics? In order to solve above questions, the high-throughput sequencing technology was applied to analyze the structural composition, diversity and function of soil bacterial communities from 700 m to 1,000 m under an identical trees (*Tilia amurensis*, which is a typical tree in these elevations). The results contribute to a better understanding of the bacterial diversity in soil and the ecological roles of bacterial communities, and assist in elucidating the driving factors shaping soil bacterial communities related to elevation in Changbai Mountains.

## Materials and methods

### Research site

This study location is within the Changbai Mountain Nature Reserve (126°55′–129°00′E; 41°23′–42°36′N) in Jilin Province, northeast of China, which belongs to a typical temperate climate with cold winters and warm summers. The average annual temperature is 4.3°C, and the average annual precipitation is 745 mm. The growing season lasts from May to September. The soil is dark brown soil developed from volcanic ash.

Four elevations were selected: 700, 800, 900, and 1,000 m above sea level, located on a north slope of Changbai Mountains. The GPS position of each elevation showed in Supplementary Table S1. The forest types between four elevations were Broad-leaved Korean Pine Forests. The dominant vegetations of 700 m were *Pinus koraiensis*, *Acer mono*, *Tilia amurensis*, *Fraxinus mandshurica*, *Quercus mongolica*, *Populus ussuriensis*. The dominant vegetations of 800 m were *P. koraiensis*, *Betula platyphylla*, *T. amurensis*, *F. mandshurica*, *Abies nephrolepis*, *Phellodendron amurense*, *Picea koraiensis.* The dominant vegetations of 800 m were *P. koraiensis*, *T. amurensis*, *Q. mongolica*, *A. nephrolepi*s, *P. jezoensis*, *A. mandshuricum*, *A. mono*, *Ulmus laciniata*, *Larix gmelinii*, *B. costata*, *P. davidiana*. The dominant vegetations of 800 m were *P. koraiensis*, *P. jezoensi*s, *A. nephrolepis*, *A. mono*, *T. amurensis*, *F. mandshurica*, *P. cathayana*, *L. gmelinii*, *A. tegmentosum*. For each elevation the above-ground vegetation was inspected and five plots were identified per elevation that all contained the same vegetation (*T. amurensis*). In October 2017, soil samples were collected at a depth of 0–20 cm with an 8 cm diameter soil auger from 10 to 15 locations along an S-shaped path. After that, the soils (10–15 soil samples) were mixed into one sample. Therefore, we finally had 5 mixed soil samples in each elevation. After removal of the surface litter and humus layer, the sampled soil sample was mixed per plot. Plant rests and coarse material was removed and the soil was homogenized and sieved through 2 mm meshes before transfer to the lab. There, the samples were divided into two parts; one was stored in the −80°C for soil microbial analysis and the other was air-dried for soil physicochemical properties analysis.

### Measurements of soil chemical properties

Soil moisture content (SMC) was determined by weight loss following desiccation. The soil organic carbon (SOC) and total nitrogen (TN) contents were determined using an elemental analyzer (Flash 2000, Thermo Fisher, Austria). The total phosphorous (TP) content was determined by the digestion method of concentrated sulfuric acid and perchloric acid treatment (Adeloju et al., 1984), and NH_4_^+^-N and NO_3_^−^-N were extracted with 2 mol·L^−1^ KCl (Miranda et al., 2001) and measured calorimetrically using an automated ion analyzer (Skalar San++, Holland, Netherlands). The contents of microbial biomass C (MBC) and N (MBN) in the soil were determined by a TOC analyzer (TOC-LCPH, Shimadzu, Japan). After mixing water and soil at a ratio of 2.5:1 (w/w), the soil pH was measured with a pH meter. Total potassium (TK) and the available potassium (AK) and the available phosphorus (AP) fraction were following a NaHCO3 extraction (Olsen et al., 1954). TK, AK and AP were determined using inductively coupled plasma atomic emission spectrometry (ICP-AES-7500, Shimadzu, Japan).

### Soil DNA extraction and Illumina MiSeq sequencing

Total DNA was extracted from the soil with an E.Z.N.A.™ Soil DNA kit (Omega Biotek, Norcross, GA, United States). The V3-V4 region of the bacterial 16S rDNA gene was amplified by PCR using bacterial universal primers 338F (5′-ACT CCT ACG GGA GGC AGC A-3′) and 806R (5′-GGA CTA CHV GGG TWT CTA AT-3′; Liu et al., 2016). Each PCR had three amplification replicates and then mixed these three replicates into one PCR mixed for sequencing. So finally there were five PCR products ready to sequence. The PCR amplification reaction was carried out using TransGen AP221-02 with TransStart Fastpfu DNA Polymerase. The total volume was 20 μl contained 4 μl 5 × FastPfu buffer, 2 μl 2.5 mmol·L^−1^ dNTPs, 1.0 μl each of 5 μmol·L^−1^ upstream primer 338F and downstream primer 806R, 0.4 μl FastPfu polymerase, 0.2 μl BSA, and 20 ng DNA template. The amplification protocol was 95°C for 5 min, followed by 33 cycles with 30s at 95°C, 20s at 55°C, and 45 s at 72°C and final extension of 10 min at 72°C. Triplicate amplification products of the same sample were mixed and checked by electrophoresis on a 2% agarose gel, excised and were recovered with a TAKARA DNA Gel Extraction kit (TAKARA Biosciences, Japan). The purified amplicons were subjected to high-throughput sequencing using the Illumina MiSeq platform. The high-throughput sequencing was performed by the Biomarker Technologies Company (Beijing China).

### Analysis of the sequencing data and statistical analysis

Sequences were analyzed using QIIME (version 1.17, http://qiime.org) software on the Biomaker bioinformation cloud platform.^1^ Forward and reverse reads were merged using the PEAR software (version 0.9.8). The merged reads were removed if the mean quality score < 20 or the length < 200 bp and the ambiguities were also removed. Chimeras were removed using Usearch software (version 7.1, https://www.drive5.com/usearch/). Exact barcode matching was implemented, which allowed for a two-nucleotide mismatch during primer matching. Operational taxonomic units (OTU) were generated at a similarity level of 97% for which the RDP (ribosomal database project) classifier Bayesian algorithm was used (Wang et al., 2007). Taxonomic analysis was performed on the representative sequences of OTU, with a confidence threshold of 0.7, and the Silva 132/16 s bacteria database was used for comparison.^2^ Before calculate the alpha diversity, we first normalized the reads according to the lowest number of reads. The alpha diversity indices Ace, Chao1, Shannon and Simpson were calculated. R software (R Development Core Team, 2016) was used to produce graphs and Principal Coordinates Analysis was used to calculate the beta diversity distance matrix based on Bray–Curtis dissimilarity (Frey et al., 2021). One-way ANOVA and Duncan’s tests were performed to detect significantly different phyla and genera and physicochemical properties for the different elevations using SPSS 22.0 (IBM SPSS Statistics for Windows). Linear discriminant analysis (LDA > 4.0) effect size (LEfSe) analysis, based on the OTU table, was performed by R software using the microeco package and this was also used for a Mantel test on the relationship of soil physicochemical parameters and soil bacterial community. A correlation heatmap for soil physicochemical properties with the most abundant 30 genera was likewise produced. The functional prediction of soil bacteria was based on the FAPROTAX code and database that were downloaded at http://www.loucalab.com/archive/FAPROTAX/lib/php/index.php?section=Download. The heatmap of functions were calculated *via* R software using “microeco” package.

## Results

### Soil physicochemical properties

Table 1 shows that no significantly differences were observed (*p* > 0.05) at different altitudes for soil pH (range: 5.48–6.15), and contents of TK (2.74–4.21 g·kg^−1^), TP (0.05–0.8 g·kg^−1^), AP (9.74–16.12 mg·kg^−1^), and SOC (10.77–17.31 g·kg^−1^). Other physical and chemical properties were significantly different (*p* < 0.05). A relatively wide range was observed for NH_4_^+^ (18.48–59.96 mg·kg^−1^) and SMC (19.17%–49.08%), while NO_3_^−^ (2.37–11.32 mg·kg^−1^), TN (11.69–33.27 g·kg^−1^) and AK (12.28–21.70 mg·kg^−1^) also significantly varied. The microbial parameters MBC also strongly varied, with a range of 55.52–510.54 mg·kg^−1^ for MBC and a range of 7.30–54.87 mg·kg^−1^ for MBN (Table 1).

**Table 1:** Soil physicochemical characteristics along an altitudinal gradient in the Changbai Mountains, Northeastern China.

Elevation (m)	pH	NH_4_^+^ (mg·kg^−1^)	NO_3_^−^ (mg·kg^−1^)	SMC (%)	TN (g·kg^−1^)	TK (g·kg^−1^)	TP (g·kg^−1^)	AK (mg·kg^−1^)	AP (mg·kg^−1^)	MBC (mg·kg^−1^)	MBN (mg·kg^−1^)	SOC (g·kg^−1^)
700	5.48 ± 0.28a	53.06 ± 7.05a	5.99 ± 1.48ab	48.19 ± 1.49a	26.64 ± 7.07ab	4.21 ± 0.47a	0.06 ± 0.01a	21.09 ± 3.29a	10.28 ± 1.93a	510.54 ± 76.55a	54.87 ± 10.44a	13.99 ± 0.97a
800	6.05 ± 0.23a	59.96 ± 5.69a	6.61 ± 2.09ab	38.52 ± 2.25b	33.27 ± 4.63a	3.44 ± 0.71a	0.08 ± 0.02a	21.70 ± 2.60a	16.12 ± 1.95a	420.52 ± 25.92a	49.12 ± 3.11b	17.31 ± 1.74a
900	5.88 ± 0.17a	18.48 ± 1.55b	2.37 ± 0.28b	19.17 ± 1.09c	11.69 ± 2.26b	2.74 ± 0.33a	0.05 ± 0.00a	12.28 ± 0.96b	9.74 ± 2.98a	55.52 ± 11.85c	7.30 ± 0.90c	10.77 ± 1.39a
1,000	6.15 ± 0.11a	54.97 ± 9.80a	11.32 ± 3.18a	44.59 ± 3.71ab	18.02 ± 8.12ab	2.74 ± 0.84a	0.07 ± 0.01a	18.21 ± 2.87ab	14.48 ± 2.20a	249.32 ± 25.54b	26.41 ± 3.81b	15.85 ± 1.22a

Values represent means ± standard deviations (*n* = 3). Different letters indicate significant (*p* < 0.05) differences between individual means assessed by one-way ANOVA followed by Tukey’s HSD *post-hoc* testing. NH_4_^+^, ammonium nitrogen; NO_3_^−^, nitrate nitrogen; SMC, soil moisture content; TN, total nitrogen; TK, Total potassium; AK, available potassium; AP, available phosphorus; MBC, Microbial biomass carbon; MBN, Microbial biomass nitrogen; SOC, soil organic carbon.

### Bacterial alpha diversity

The variable region of bacterial 16S rDNA was amplified from DNA isolated from the soil samples and sequenced to compare the bacterial communities that were present (Table 2). From the obtained sequences, diversity indices were calculated (Table 2). The Shannon index did not vary significantly between the four elevation gradients (*p* > 0.05), but the abundance indices (Richness, Chao1, and ACE index) all varied significantly (*p* < 0.05). The Chao1 index showed significant differences at 900 m and 1,000 m, whereas no differences were observed at other altitudes; the ACE index was significant different at 900 m and 1,000 m, and the Richness index differed between 700 and 900 m (Table 2).

**Table 2:** Bacterial α diversity in the soil collected at different altitudes.

Elevation (m)	Richness	Chao1	ACE	Shannon
700	1,666 ± 51.3b	1739.9 ± 18.9ab	1726.5 ± 16.90b	6.4 ± 0.06a
800	1707 ± 28.6ab	1758.1 ± 13.6ab	1746.7 ± 13.0ab	6.4 ± 0.02a
900	1738 ± 18.3a	1787.4 ± 8.6a	1776.4 ± 8.3a	6.45 ± 0.02a
1,000	1,680 ± 44.8bab	1746.37 ± 13.2b	1728.4 ± 15.1b	6.41 ± 0.03a

Values represent means ± standard deviations (*n* = 5). Different letters indicate significant (*p* < 0.05) differences between individual means assessed by one-way ANOVA followed by Tukey’s HSD *post-hoc* testing.

Correlation analysis between these indices and the soil physicochemical properties revealed a strongly significant positive correlation between the Shannon index and soil pH (Supplementary Table S2, *p* < 0.01), while the Richness, ACE and Chao1 indices were strongly negatively correlated with soil NH_4_^+^, and SMC (*p* < 0.01). Significant negative correlations were also observed between these three indices and MBC (*p* < 0.05, Supplementary Table S2).

### Bacterial beta diversity

**Figure 1:**
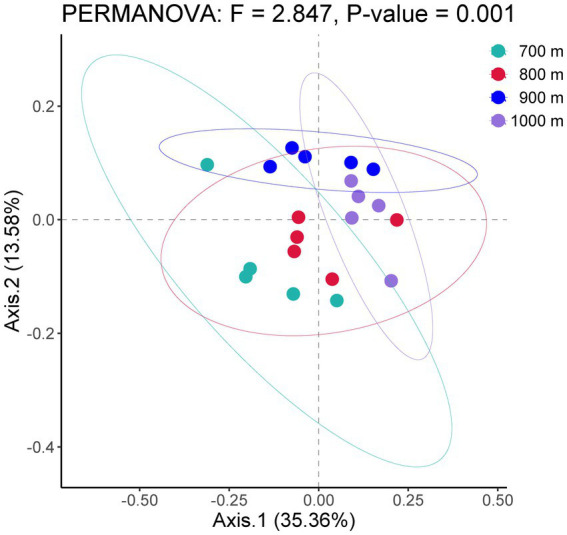
Principal coordinates analysis of bacterial communities in the soil at four altitudes with similar vegetation cover in Changbai Mountains, Northeastern China.

The bacterial community structure along the altitudinal gradient was graphically compared by Principal Coordinates Analysis (Figure 1). The five soil samples per altitude were separately analyzed. The ordination showed that the bacterial communities were clearly separated by altitude, and indeed the PERMANOVA analysis of the bacterial community among 700 m, 800 m, 900 m and 1,000 m was significantly different (*F* = 2.847, *p* < 0.01).

### Composition of the soil bacterial community

**Figure 2:**
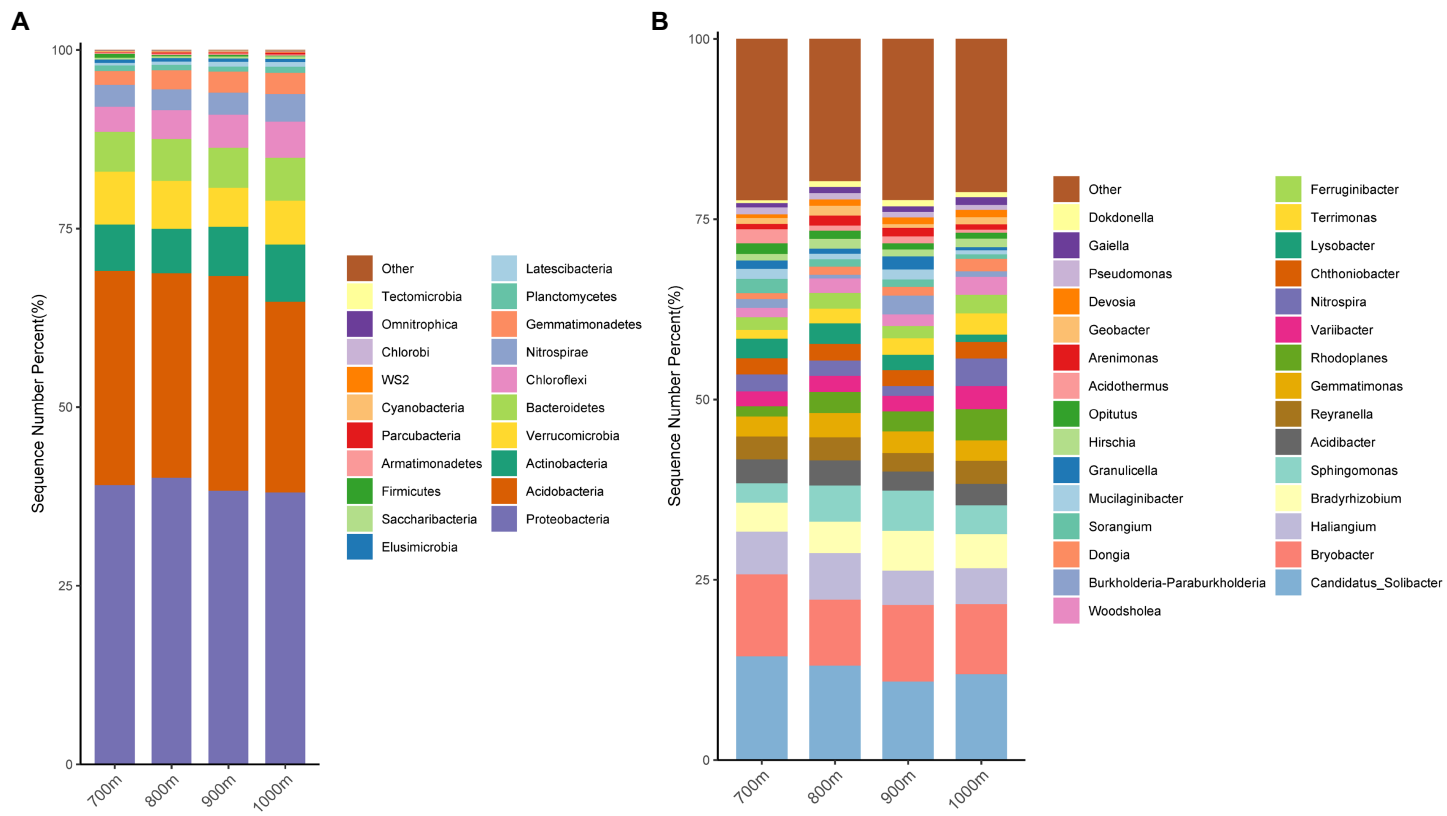
Relative abundance of the dominant bacterial phyla **(A)**, and genera **(B)**, in soils collected at the four indicated altitudes in the Changbai Mountains, Northeastern China.

The obtained sequences were attributed to OTUs to investigate the bacterial community composition. A total of 52 bacterial phyla were identified; sequences that could not be classified to a known phylum were collectively reported as ‘others’ (Figure 2A). The highest relative abundance (r.a.) was observed for Proteobacteria. Other phyla reaching r.a. > 1% were, in descending order: Acidobacteria, Actinobacteria, Verrucomicrobia, Bacteroidetes, Chloroflexi, Nitrospirae, Gemmatimonadetes, and Planctomycetes, and this order did not vary with altitude, although variation in r.a. between the elevations was observed (Figure 2A). The r.a. of the 8 phyla (relative abundance > 0.1%) at each altitude is summarized in Table 3. Significant differences in r.a. between the four elevations was found for the phyla Gemmatimonadetes and Latescibacteria (highest r.a. at 900 m and lowest at 700 m for both), Firmicutes (highest at 900 m and lowest at 1,000 m), Parcubacteria, Actinobacteria and WS2 (highest at 1,000 m and lowest at 700 m for all three; Supplementary Figure S1, *p* < 0.05). The r.a. of the other phyla listed in Table 3 did not differ significantly with altitude (*p* > 0.05).

**Table 3:** Relative abundance of the dominant bacterial phyla (top) and genera (bottom) in the soil along an altitudinal gradient in the Changbai Mountains, northeastern China.

Phylum	700 m	800 m	900 m	1,000 m	*p*
Proteobacteria	39.1%	40.0%	38.3%	38.0%	
Acidobacteria	30.0%	28.7%	30.0%	26.8%	
Verrucomicrobia	7.4%	6.8%	5.4%	6.2%	
Actinobacteria	6.5%	6.2%	6.9%	8.0%	<0.05
Bacteroidetes	5.6%	5.8%	5.6%	6.0%	
Chloroflexi	3.5%	4.0%	4.6%	5.1%	
Nitrospirae	3.1%	2.9%	3.1%	3.9%	
Gemmatimonadetes	1.9%	2.7%	2.9%	3.0%	<0.05
**Genus (>1%)**					
*Variibacter*	2.0%	2.2%	2.2%	3.2%	<0.05
*Rhodoplanes*	1.4%	2.9%	2.7%	4.4%	<0.05
*Lysobacter*	2.7%	2.9%	2.2%	1.0%	<0.05
*Woodsholea*	1.2%	2.0%	1.6%	2.5%	<0.05
*Dongia*	0.8%	1.1%	1.2%	1.7%	<0.05
*Terrimonas*	1.1%	2.0%	2.2%	2.9%	<0.05
*Sorangium*	2.0%	1.0%	1.0%	0.6%	<0.05
*Nitrospira*	2.1%	2.1%	1.3%	3.7%	<0.05

The sequences were also analyzed at the genus level (Figure 2B). For all four altitudes, the most abundant genera were (in descending order) *Candidatus Solibacter*, *Bryobacter*, *Haliangium*, *Bradyrhizobium*, *Sphingomonas*, *Acidibacter*, *Reyranella*, *Gemmatimonas*, *Rhodoplanes*, and *Variibacter*, which in combination accounted for 50% or more of the identified genera (Figure 2B). Ten more genera reached a relative abundance >1% (Figure 2B). Statistically significant differences (*p* < 0.05) in r.a. between the altitudes was observed for the genera *Variibacter*, *Rhodoplanes*, *Lysobacter*, *Woodsholea*, *Dongia*, *Terrimonas*, *Sorangium*, and *Nitrospira* (Table 3, *p* < 0.05).

**Figure 3:**
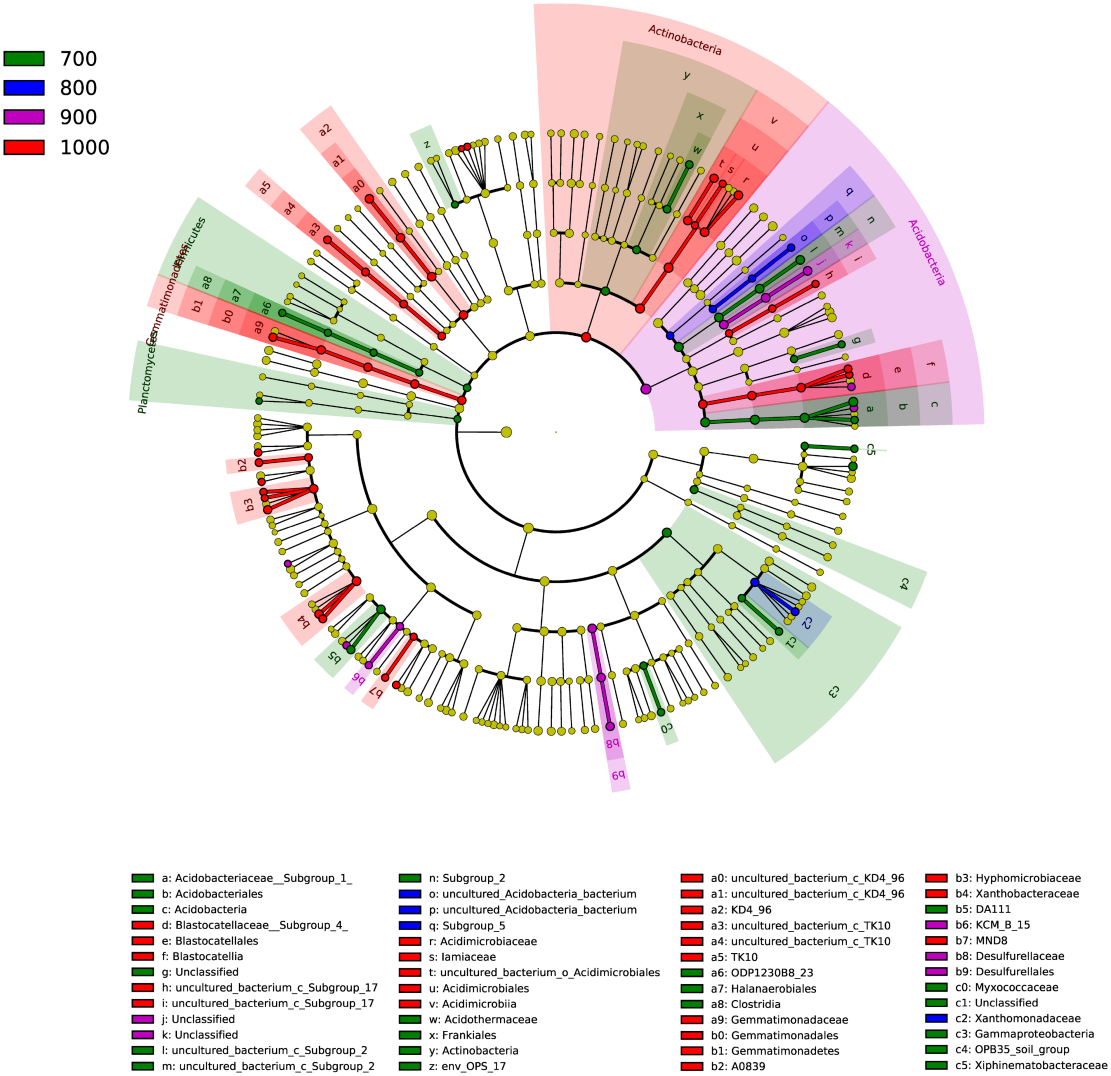
Cladogram of the soil bacterial communities with LDA > 4.0 in the four elevations. The circular plot shows bacterial taxa from phyla to genera starting from the center.

A total of 52 genera were identified in the four elevations (Figure 3), as indicated by LDA effect size scores of >4.0. Indicator genera were identified: *Acidothermus* for 700 m, *Lysobacter* for 800 m, *Bauldia* and *Granulicella* for 900 m and *Rhodoplanes*, *Variibacter, Dongia*, and *Woodsholea* were indicator genera for 1,000 m.

### Relationship between bacterial community structure and soil physicochemical properties

A Mantel analysis of soil bacterial OTU levels and environmental factors showed that pH was the main soil factor affecting the bacterial communities (Table 3, *p* < 0.05), with differences noted at the different altitudes: strong correlations were seen at 800 m and 900 m for pH (*p* < 0.01) but the correlation was weaker at 1,000 m (*p* < 0.05) and not significant at 200 m.

**Figure 4:**
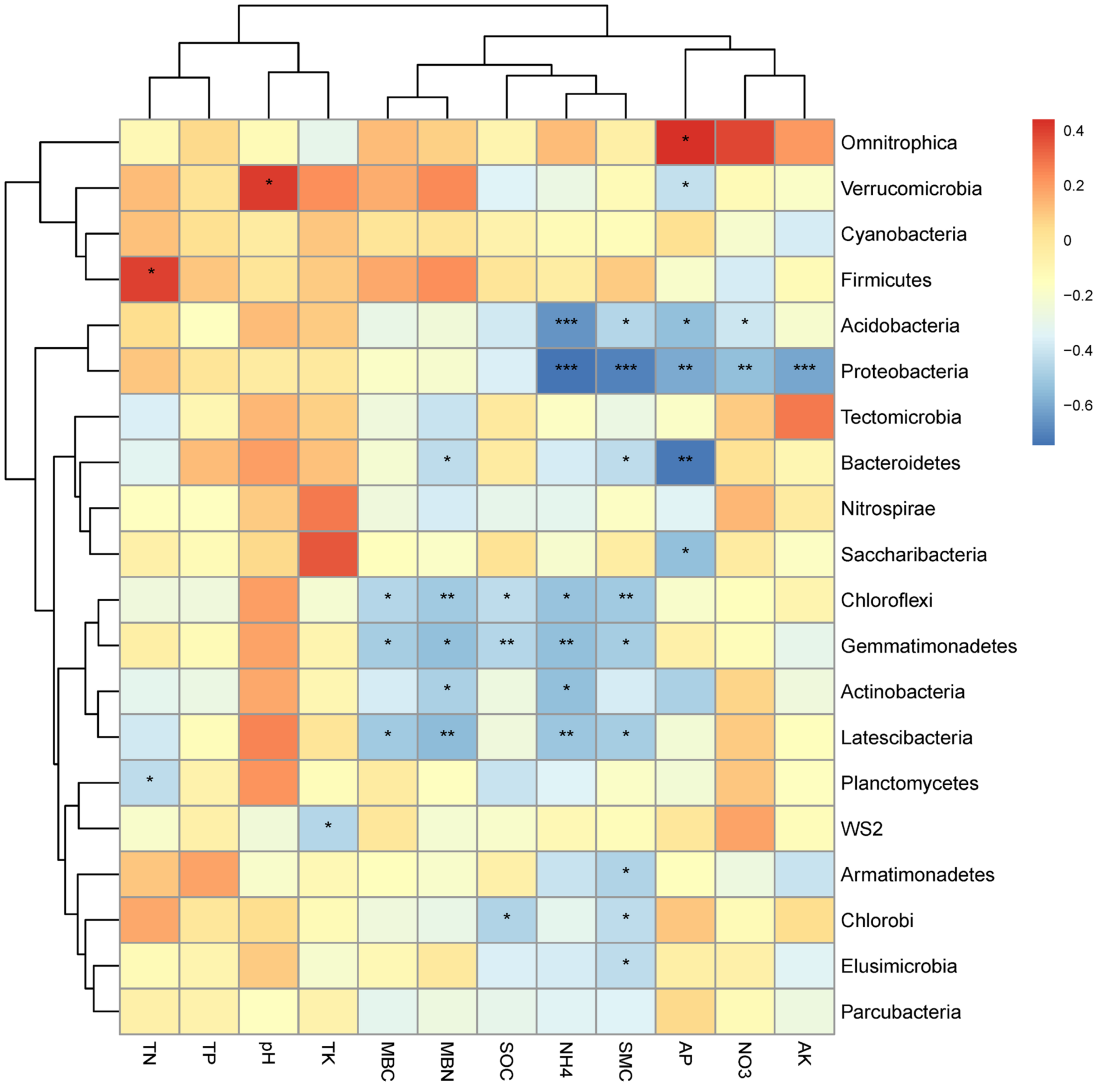
Heat map of the correlations between soil physicochemical properties and the top 20 bacterial phyla, with significance indicated as * at the 0.05 level (one-tailed) and ** at the 0.01 level (two-tailed).

The correlations between the relative abundance of the top 20 bacterial phyla and the soil physicochemical properties for all altitudes combined is graphically summarized in a heat map (Figure 4). A strong positive correlation was observed between Verrucomicrobia and pH, Fircumicutes and TN, as well as Omnitrophica and AP (Figure 4). Strong negative correlations were observed for Proteobacteria and NH_4_^+^, SMC and AK together with weaker correlation for AP and NO_3_^−^. Strong negative correlations were also observed for Bacteroidetes with AP. Acidobacteria correlated strongly negatively with NH_4_^+^ and AP. Weaker negative correlations were observed for Chloroflexi with soil MBN, and SMC for Gemmatimonadetes with SOC and NH_4_^+^; for Latescibacteria with MBN, and NH_4_^+^. Weaker positively correlations were observed for Fimicutes with soil TN, and Verrucomicrobia with pH.

The correlations between genera and the soil characteristics are summarized in Supplementary Figure S1. The soil factors affecting relative abundance of multiple genera were pH, MBC, MBN, NO_3_^−^, AK, and SMC, and these produced positive or negative correlations, depending on the genus (Supplementary Figure S1). TK was the only factor correlating with a single genus (*Pedobacter*). The correlation patterns for the various genera of NO_3_^−^, NH_4_^+^, AK, and SMC clustered together, as did the patterns for MBC and MBN (Supplementary Figure S1).

### Inferred functionality of soil bacteria and their differences between different altitudes

**Figure 5:**
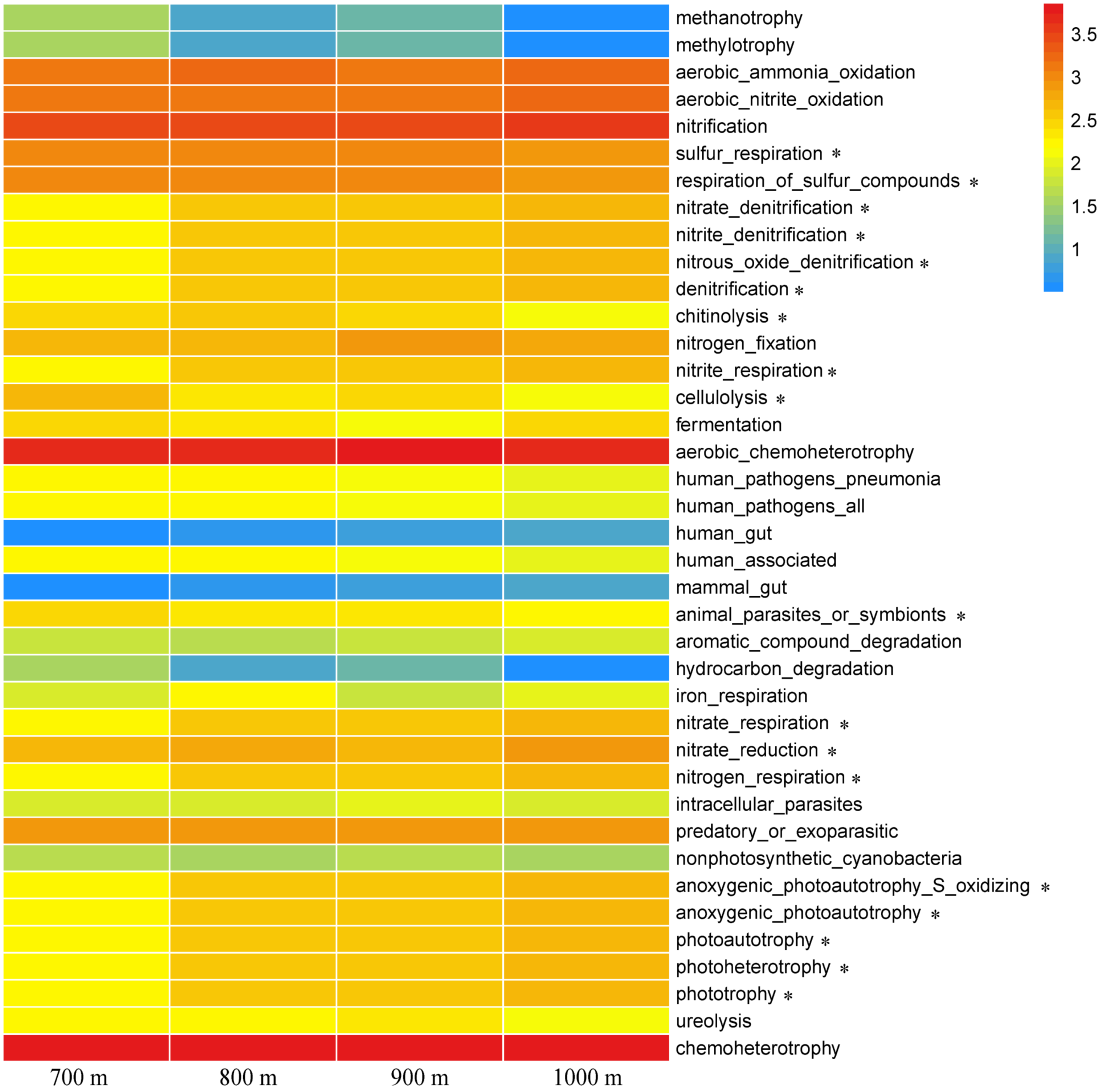
Heatmap of functional processes inferred from the relative abundance of the soil bacteria at the five different elevations. The color gradient (red, yellow, and blue) represents the relative abundance of the inferred bacterial functions from high to low. * indicated difference at the 0.05 level based on one-way ANOVA.

FAPROTAX analysis was used to infer a total of 39 functional groups, and their relative abundance at the different altitudes were compared in a heatmap (Figure 5). Chemoheterotrophy was the most dominant functional category, followed by aerobic chemoheterotrophy and nitrification, but they did not correlate with altitude as they were found across the elevation gradient (*p* > 0.05, Figure 5). Many functional categories identified in the microbial communities of the soil collected at 800 m and 900 m resembled each other, while those at 700 m was quite distinct. As expected, trends along the elevation gradient were similar for the functions of nitrate denitrification, nitrite denitrification, nitrous oxide denitrification, denitrification, nitrate_respiration, and nitrogen respiration, as these are all involved in related N metabolic processes. Those trends resembled the trends for anoxygenic photoautotrophy S oxidizing, anoxygenic photoautotrophy, photoautotrophy, photoheterotrophy, and phototrophy. Other functions that varied along the gradient were sulfur_respiration/respiration of sulfur compounds, chitinolysis, nitrite respiration, cellulolysis, animal parasite or symbionts, and nitrate reduction (*p* < 0.05, Figure 5).

## Discussion

### Effects of elevation on bacterial alpha diversity

A number of studies have suggested that specific soil microbial communities exist at specific altitudes (Liu et al., 2013; Shen et al., 2013; Wang et al., 2015). The present study showed that soil bacterial abundance changed under different altitude gradients, but the diversity of the community did not change (Figure 1). In our study, the above-ground vegetation did not vary between altitudes, which might explain why the soil bacterial Shannon index did not change significantly. Further, at the investigated elevation between 700 and 1,000 m, elevation may not be the main driver of Shannon diversity in soil bacteria. The observed changes in ACE and Chao richness indices may be attributed to the effect of soil physicochemical factors. Our results show that SMC, NH_4_^+^ and MBC significantly and negatively correlated with soil ACE and Chao1 indices. This indicates elevation can affect changes in soil physicochemical factors that influence the abundance of bacteria. Han et al. (2018) studied the diversity of soil bacteria between 699 and 1,177 m in broadleaf Korean pine forests in Changbai Mountains and reported no change in the Shannon diversity index, which is consistent with the results of this study: we found that altitude did not lead to changes in Shannon diversity of soil bacteria from 700 to 1,000 m altitude. However, whereas we detected variation in ACE and Chao indices, Han et al. (2018) did not observe such an effect. The discrepancy may be explained by soil physicochemical factors that remained unaltered in their comparison, as Ping et al. (2017) showed that soil TOC, TN, SMC, and pH were not significantly altered under broadleaf red pine forests at 699–1,044 m. Shen et al. (2013) found that soil bacterial PD diversity indices and OTUs did not change at 530–760 m in Changbai Mountains, probably because the low altitude scale did not significantly affect bacterial diversity. Our study only covered a difference of 400 m, which may also account for the fact that soil bacterial diversity was not significantly altered. Moreover, it is worth noting that Han et al. (2015) found a significant change in the functional Shannon diversity of soil bacteria at 699–1,177 m using the Biolog-Eco technique. This suggests that different technical tools may also have some influence on the results. Above studies showed that the above-vegetation and the analysis method would have the different results of soil microorganism. Collectively, the distribution of soil bacterial diversity and composition along the altitudes is complex and bacterial communities respond to different driving processes at the altitudinal scale (Shen et al., 2019). The trend of the soil ACE abundance index along the altitude was also not consistent with Shannon, suggesting that a uniform pattern of bacterial diversity may be difficult to achieve along the altitude gradient.

### Bacterial compositions in different altitudes in Changbai Mountains

Our study identified a number of dominant bacterial phyla whose relative abundance differed at different elevations, although they were not necessarily present in high abundance (Figure 2A, Table 3). The already mentioned study by Han et al. (2018) described as bacterial phyla Acidobacteria, Proteobacteria, Actinobacteria Verrucomicrobia, and Chloroflexi, which was mostly consistent with our results, but they identified Acidobacteria to differ at different altitudes, which is not what we found. Instead, among the dominant bacterial phyla we detected (>3% relative abundance), only Actinobacteria significantly varied (Table 3). Although the elevation gradients of both studies were similar, Han et al. (2018) did not consider the influence of aboveground vegetation. Soil bacteria are not only affected by changes in soil microhabitats caused by the elevation gradient, but also by aboveground plant diversity. The soil phylum acidobacteria, a relatively high abundant phylum in soil, may change due to the diversification of the composition of above-ground plant diversity. In the present study, the main soil bacterial phyla, such as Acidobacteria and Proteobacteria, did not change significantly because the above-ground vegetation was the same and therefore the effects caused by above-ground vegetation and litter could be ignored. As changes in soil bacterial species under the altitude gradient are also influenced by above-ground vegetation, studies in which above-ground vegetation is not uniform are difficult to interpret and compare.

A number of dominant genera were also detected at significantly different abundances along the altitude gradient, including *Variibacter*, *Rhodoplanes*, *Lysobacter*, *Woodsholea*, *Dongia*, *Terrimonas*, and *Sorangium*, while the most abundant genera were *Candidatus_Solibacter*, *Bryobacter*, *Haliangium*, *Bradyrhizobium*, *Sphingomonas*, *Acidibacter* (Figure 2B; Table 3). Surprisingly, in the comparable study by Han et al. (2018) the dominant bacterial genera were *DA101*_*soil*_*group*_*norank*, *Xanthobacteraceae*_*uncultured*, *Subgroup*_*6*_*norank* and *Bradyrhizobium* while Li et al. (2017) reported *Devosia*, *Dokdonella*, *Phaselicystis*, *Rhodobacter*, and *Conexibacter* as the main genera in the inter-rhizosphere soil community at 2,000 m of Changbai Mountain. This indicates a strong variation in composition of soil bacteria at the genus level between different locations and studies, even within the same area. Differences in temperature, soil nutrients and plant composition related to altitude all contribute to the distribution of soil bacteria, and differences become more apparent at lower taxonomic levels.

### The relationships of bacterial structure and soil physicochemical properties in different altitudes

The soil bacterial community structure composition is regulated by soil physicochemical factors. The results of the mantel analysis in this study showed that soil physicochemical properties were significantly correlated with soil bacterial community structure (Table 3). The soil bacterial community structure at different altitude gradients was influenced by different soil physicochemical properties. At 700 m and 800 m, the soil bacterial community structure was correlated with MBC, MBN, TN, AK, TK, TP, AP, NO_3_^−^, MC, and NH_4_^+^; pH showed a positive correlation with the soil bacterial community structure at 1,000 m (Table 3). This indicated that the structural composition of the soil bacterial community is influenced by soil physicochemical factors and that the driving factors for soil bacteria are not the same at different altitudes. In mountain forest ecosystems, altitude can lead to dramatic changes in climatic factors such as temperature and precipitation (Chen et al., 2011), and microorganisms are extremely sensitive to environmental changes. Xia et al. (2022) found that soil microbial community structure was positively correlated with soil SOC and TN. This is inconsistent to the results of our study. Tan et al. (2013) found that changes in soil TP and AP levels affected the composition of soil bacterial communities. Shen et al. (2013) showed that soil microorganisms were most significantly influenced by pH. This indicated that the structure and diversity of soil microbial communities in a given area were influenced by a combination of factors (Liu et al., 2010).

In addition, according to Pearson correlation analyses, the 20 most abundant bacterial phyla were mainly influenced by NH_4_^+^-N, NO_3_^−^-N, TK, MBC, MBN, TC, MC (Figure 4), and Proteobacteria, Acidobacteria, Bacteroidetes, Chlroflexi, Gemmatimonadetes, Latescibacteria, Actinobacteria, Planctomycetes, Armatimonadetes, Elusimicrobia, and Chlorobi all showed significantly negative correlations with soil nutrients. This is inconsistent with previous studies, for example Proteobacteria are eutrophic microorganisms, which have been shown a positive correlation with soil nutrients (He et al., 2021). This may be because at the bacterial phylum level, not all Proteobacteria may be constrained by nutrient content when nutrient availability is adequate. The Changbai Mountains is a volcanic mountain ecosystem (Zhang et al., 2022) with sufficient nutrient content to provide for the growth of Proteobacteria. It is also worth noting that our study was carried out on the composition of the soil bacterial community under *T. amurensis*, which may have affected the bacterial distribution. The mechanisms regulating the abundance of soil microorganisms under the trees need to be further investigated.

### Changes in soil bacterial function under purple linden trees at different altitudes

FAPROTAX is an effective tool to predict the soil bacterial function (Louca et al., 2016), and this tool revealed bacterial functional groups that changed significantly with different elevations (Figure 5). It can be expected that changes in bacterial composition influence soil carbon and nutrient functions (Frey et al., 2016). Moreover, phototrophy, nitrification, nitrate respiration, and nitrite respiration play crucial functions in soil carbon and nitrogen cycling in 800–1,000 m altitudes, while functions related to parasites and cellulolysis and sulfur respiration may be more important in 700 m altitudes. Altitudes changes usually alter soil physicochemical properties, which sets broadleaf forests apart from conifer forests. Therefore, because of variation in soil nutrient contents, the soil bacterial functions may differ significantly between the four altitudes.

## Conclusion

Soil bacterial richness differed significantly, but Shannon diversity did not change under same above-ground vegetation in Changbai Mountains, which supports our 1^st^ hypothesis. Soil dominant phyla relative abundance were affected by soil nutrient (i.e., SMC, SOC, MBN, MBC, AP, NH_4,_ and NO_3_), but the dominant genera relative abundance were affected by pH, MBC, MBN, NO_3_, AK, and SMC. This showed that the composition of soil bacterial has different correlations with soil physicochemical parameters, which supports our 2nd hypothesis. This comprehensive study to characterize bacterial communities and their diversities at different elevations under same above-ground vegetation in Changbai Mountains revealed that the soil physiochemical characteristics have a significant effect on the overall diversity of bacterial communities along the elevation gradient, but from the available data it is difficult to predict overall bacterial diversity along elevation. Nevertheless, this study contributes to the understanding of bacterial community composition and diversity patterns at local altitudinal scales, and provides a theoretical basis for predicting the response, adaptation and feedback of microbial communities to changes in environmental conditions.

## Data availability statement

The data presented in the study are deposited in the Sequence Read Archive repository, accession number PRJNA888966.

## Author contributions

ML designed and performed the experiment and prepared this manuscript. GD helped to collect the soil samples and revised this manuscript. LM designed this experiment. All authors contributed to the article and approved the submitted version.

## Funding

This work was supported by the Ministry of Science and Technology Fundamental Resources Investigation Project of China (2019FY100505).

## Conflict of interest

The authors declare that the research was conducted in the absence of any commercial or financial relationships that could be construed as a potential conflict of interest.

## Publisher’s note

All claims expressed in this article are solely those of the authors and do not necessarily represent those of their affiliated organizations, or those of the publisher, the editors and the reviewers. Any product that may be evaluated in this article, or claim that may be made by its manufacturer, is not guaranteed or endorsed by the publisher.

## Footnotes

^1^ https://international.biocloud.net

^2^ https://www.arb-silva.de/
