# Supplementary material for: Composition and diversity of soil bacterial communities under identical vegetation along an elevational gradient in Changbai Mountains, China
Source: Front Microbiol. 2022 Dec 1;13:1065412. doi: 10.3389/fmicb.2022.1065412 (PMC9751831; doi:10.3389/fmicb.2022.1065412)
Supplement: Supplementary file 1 [file Table_1.doc]

Supplementary Material

## Supplementary Tables

**Table S1.** The position of each elevation in Changbai Mountains, China.

| **Altitudes (m)** | **Latitude** | **Longitude** |
| --- | --- | --- |
| 700 | 42°34'30'' | 127°56'20'' |
| 800 | 42°20'48'' | 128°05'30'' |
| 900 | 42°18'20'' | 128°07'40'' |
| 1000 | 42°15'50'' | 128°09'25'' |
