# Supplementary material for: Composition and diversity of soil bacterial communities under identical vegetation along an elevational gradient in Changbai Mountains, China
Source: Front Microbiol. 2022 Dec 1;13:1065412. doi: 10.3389/fmicb.2022.1065412 (PMC9751831; doi:10.3389/fmicb.2022.1065412)
Supplement: Supplementary file 2 [file Table_2.doc]

Supplementary Material

## Supplementary Tables

**Table S2.** Correlation analysis of soil bacterial α diversity and soil physicochemical properties in the Changbai Mountains, northeastern China, for the four investigated elevations combined.

|  | Richness | ACE | Chao1 | Shannon |
| --- | --- | --- | --- | --- |
| pH | 0.18 | 0.06 | 0.12 | 0.60** |
| NH_4_^+^ | -0.64** | -0.71** | -0.68** | -0.12 |
| NO_3_^-^ | -0.06 | -0.08 | 0.01 | 0.18 |
| SMC | -0.62** | -0.63** | -0.58** | -0.19 |
| TN | -0.23 | -0.19 | -0.24 | -0.36 |
| TK | -0.23 | -0.26 | -0.27 | 0.03 |
| TP | 0.05 | -0.12 | -0.18 | 0.17 |
| AK | -0.33 | -0.35 | -0.30 | 0.16 |
| AP | 0.07 | 0.03 | 0.08 | 0.19 |
| MBC | -0.54* | -0.48* | -0.49* | -0.31 |
| MBN | -0.39 | -0.32 | -0.35 | -0.35 |
| SOC | -0.32 | -0.26 | -0.17 | -0.20 |

Statistical significance is indicated as * (*P*<0.05) and ** (*P*<0.01).
