# Supplementary figures and images for: Composition and diversity of soil bacterial communities under identical vegetation along an elevational gradient in Changbai Mountains, China
Source: Front Microbiol. 2022 Dec 1;13:1065412. doi: 10.3389/fmicb.2022.1065412 (PMC9751831; doi:10.3389/fmicb.2022.1065412)

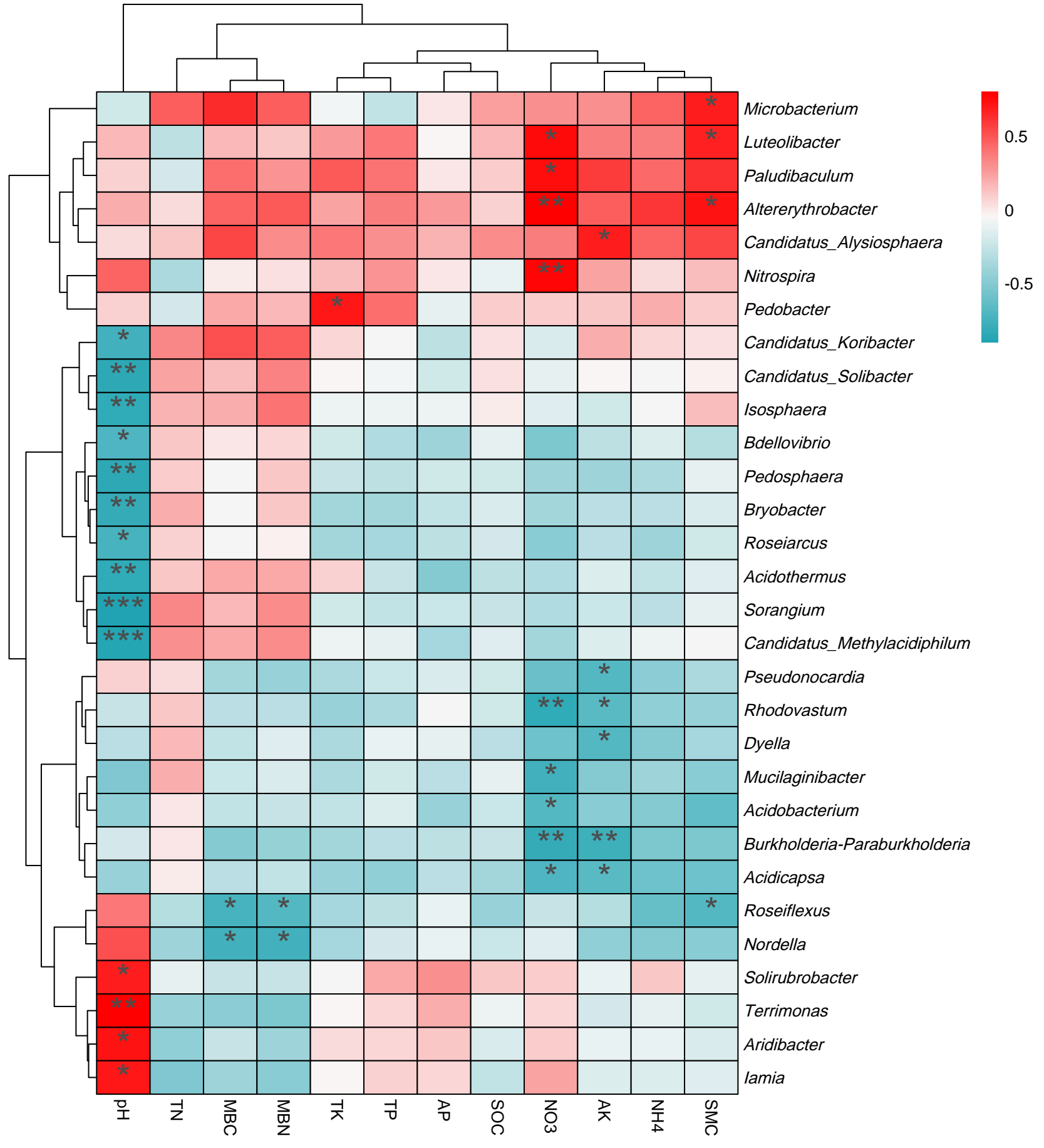

Supplement: Supplementary file 3 [file Image_1.pdf]
